# Surprising Complexity of the [Gd(AAZTA)(H_2_O)_2_]^−^ Chelate Revealed by NMR in the Frequency and Time Domains

**DOI:** 10.1021/acs.inorgchem.1c03194

**Published:** 2021-12-10

**Authors:** Daniela Lalli, Fabio Carniato, Lorenzo Tei, Carlos Platas-Iglesias, Mauro Botta

**Affiliations:** † Dipartimento di Scienze e Innovazione Tecnologica, Università del Piemonte Orientale “A. Avogadro”, Viale T. Michel 11, 15121 Alessandria, Italy; ‡ Centro de Investigacións Científicas Avanzadas (CICA) and Departamento de Química, Facultade de Ciencias, Universidade da Coruña, 15071 A Coruña, Galicia, Spain; § Magnetic Resonance Platform (PRISMA-UPO), Università del Piemonte Orientale “A. Avogadro”, Viale T. Michel 11, 15121 Alessandria, Italy

## Abstract

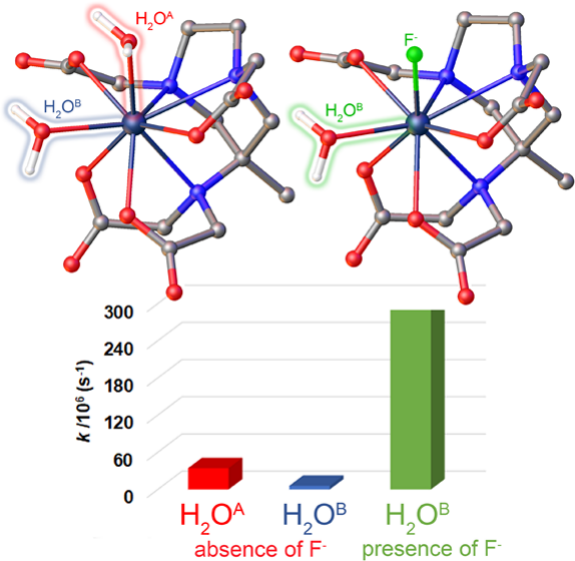

Typically, Ln(III) complexes are isostructural along the series, which enables studying one particular metal chelate to derive the structural features of the others. This is not the case for [Ln(AAZTA)(H_2_O)*_x_*]^−^ (*x* = 1, 2) systems, where structural variations along the series cause changes in the hydration number of the different metal complexes, and in particular the loss of one of the two metal-coordinated water molecules between Ho and Er. Herein, we present a ^1^H field-cycling relaxometry and ^17^O NMR study that enables accessing the different exchange dynamics processes involving the two water molecules bound to the metal center in the [Gd(AAZTA)(H_2_O)_2_]^−^ complex. The resulting picture shows one Gd-bound water molecule with an exchange rate ∼6 times faster than that of the other, due to a longer metal–water distance, in accordance with density functional theory (DFT) calculations. The substitution of the more labile water molecule with a fluoride anion in a diamagnetic-isostructural analogue of the Gd-complex, [Y(AAZTA)(H_2_O)_2_]^−^, allows us to follow the chemical exchange process by high-resolution NMR and to describe its thermodynamic behavior. Taken together, the variety of tools offered by NMR (including high-resolution ^1^H, ^19^F NMR as a function of temperature, ^1^H longitudinal relaxation rates vs *B*_0_, and ^17^O transverse relaxation rates vs *T*) provides a complete description of the structure and exchange dynamics of these Ln-complexes along the series.

## Abstract 2

Herein, we present a ^1^H field−cycling relaxometry and ^17^O NMR study that enables accessing the different exchange dynamics processes involving the two water molecules bound to the metal center in the [Gd(AAZTA)(H_2_O)_2_]^−^ complex.

## Introduction

Magnetic resonance imaging (MRI) has rapidly emerged as one of the most important and widespread tools in diagnostic clinical medicine and biomedical preclinical research. This is due to several favorable properties that characterize the technique, including the great spatial resolution, typically of the order of millimeters in the clinical setting, the ability to produce cross-sectional images of the body with excellent soft-tissue contrast, and the absence of ionizing radiation such as those used in X-ray and computed tomography (CT) scan. Commonly, the various imaging techniques are associated with the use of suitable contrast media, which have the purpose of improving the signal-to-noise ratio and optimizing the visualization of the morphology and physiology. Despite the remarkable level of intrinsic contrast of MRI images, even this diagnostic modality largely uses contrast agents that, as it is well known, are based on Gd(III) complexes.^1−3^ These exogenous probes possess high efficacy in accelerating the longitudinal relaxation rate (*R*_1_) of the water protons of the tissues in which they are distributed; therefore, they allow shortening of the measurement time and improvement of the contrast-to-noise ratio of MR images, thus facilitating the overall diagnosis. The clinically approved gadolinium(III)-based contrast agents (GBCAs) are complexes with octadentate polyamino–polycarboxylate ligands in which the metal ion completes its coordination number (CN = 9) by binding to one water molecule (*q* = 1).^4−6^ The high thermodynamic stability, kinetic inertness, and fast clearance of such complexes, which are essential prerequisites for their use in clinics, represent additional important benefits. Despite the many advantages associated with the use of GBCAs, their relaxivity values in clinical fields are only a fraction of those theoretically achievable.^7^ To compensate for their low efficacy, contrast agents are typically administered in relatively high doses to gain high-quality MRI images.^3^ On the other hand, the development of new GBCAs with enhanced efficacy, high thermodynamic stability, and kinetic inertness would allow reducing the injected doses. This would have a beneficial impact in terms of costs and could contribute to minimizing the possibility of long-term Gd deposition. The efficacy of a GBCA is associated with the relaxivity parameter (*r*_1_), which measures the *R*_1_ increase of the water protons per millimolar unit concentration of the paramagnetic ion and, for clinically used CAs, is about 5 mM^–1^ s^–1^ (at 1.5 T and 298 K).^1,7^ For this reason, the research toward the optimization of the values of *r*_1_ has been very active in the last 30 years and has led to an in-depth knowledge of the relationship between *r*_1_ and the molecular parameters that describe the structural aspects and dynamic processes in which the complexes are involved.

At the magnetic field values of clinical and preclinical relevance (approx. 1–7 T), *r*_1_ is essentially determined by the molecular tumbling rate (τ_R_), by the hydration state (*q*), by the average lifetime (τ_M_) of the coordinated water molecules, and by the electronic relaxation of the Gd^3+^ ion (described by the parameters Δ^2^ and τ_V_).^1,2^

**Scheme 1:**
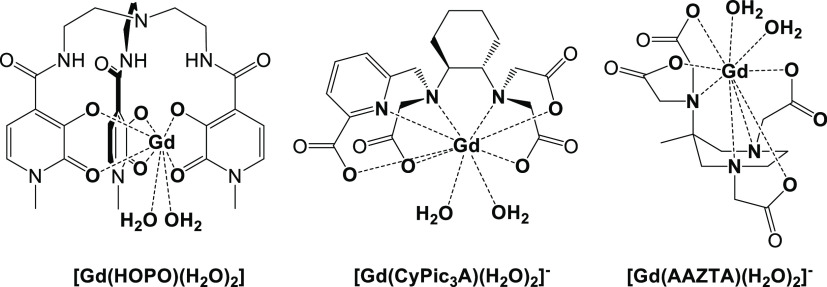
Chemical Structure of the Complexes Described in This Study

One of the possible ways to increase the efficiency of CAs is to increase the number of metal-bound water molecules, which can be achieved by the use of heptadentate ligands. However, the loss of one donor atom from the ligand comes at the cost of decreased thermodynamic stability and/or kinetic inertness. In addition, shortly after the initial studies, it was found that most of the *q* > 1 complexes show a pronounced tendency to form ternary complexes with oxyanions of biological relevance (e.g., carbonate, lactate, malonate, or oxalate) with the displacement of one or both water molecules.^8,9^ As a primary consequence, this is accompanied by a marked decrease in relaxivity, an effect opposite to the one sought. This process likely represents one of the steps involved in the transmetallation reaction in which Gd^3+^ is replaced by other endogenous metal ions (mostly Zn^2+^ and Cu^2+^) under physiological conditions. Over the years, only a small number of complexes with *q* = 2 that do not strictly follow this behavior and feature improved properties have been reported.^10,11^ The family of hydroxypyridinone (HOPO)-based complexes (Scheme 1), developed by Raymond et al. since the mid-1990s, is a notable example. The ligands are hexadentate, leaving two open sites for water coordination in the eight-coordinate Gd-complexes.^12,13^ The complexes have considerable thermodynamic stability, high *r*_1_ values, and rapid exchange of the bound water. Furthermore, they do not readily form ternary complexes with coordinating anions.

Similar properties are found in the Gd-complex with an open-chain ligand containing three acetate arms and one picolinate group, reported by Caravan et al. (Scheme 1).^14^ Finally, a third class of *q* = 2 compounds is represented by the GdAAZTA-like complexes (Scheme 1).^15^ AAZTA and derivatives are mesocyclic heptadentate ligands that form complexes with the Gd^3+^ ions that are not only thermodynamically stable but also kinetically inert.^16,17^ [Gd(AAZTA)(H_2_O)_2_]^−^ represents an excellent candidate for *in vivo* applications in preclinical studies, due to several favorable properties: easy and cheap to synthesize, it shows high thermodynamic stability, high relaxivity, and two inner-sphere water molecules in sufficiently fast water exchange with the bulk that are not displaced by bidentate anions in physiological media. Moreover, near physiological conditions, [Gd(AAZTA)(H_2_O)_2_]^−^ is significantly more kinetically inert than GdDTPA.^15,16^

Despite AAZTA being reported in 2004,^15^ only recently it has been shown that when bound to different members of the Ln series, the resulting complexes display unexpected and unique chemical properties. A decrease of the hydration state (*q* changes from 2 to 1) is observed along the Ln series, which is surprisingly accompanied by a remarkable decrease of the water exchange rate, ca. 2–3 orders of magnitude.^18^ Near the end of the series, the coordinated water molecule becomes so tightly bound to the metal, that its signal can be directly detected in high-resolution ^1^H NMR spectra of the Yb(III)-complex.^19^ The residence lifetime of the bound water molecule, measured by chemical exchange saturation transfer (CEST) experiments (150 μs), is 4 orders of magnitude higher than that of [Gd(AAZTA)(H_2_O)_2_]^−^. More recently, a systematic study on the Ln(III) complexes with AAZTA highlighted that the structural change occurs along the Ln series between Ho and Er, due to the increased steric compression of the ligand on the bound water molecules upon decreasing the metal ion size.^20^ Most importantly, two coordinated water molecules in the [Ho(AAZTA)(H_2_O)_2_]^−^ complex were detected by CEST experiments near 0 °C, which resonate at different chemical shift values (−47 and −255 ppm) and are characterized by significantly different water exchange rates (*k*_ex_ = 1/τ_M_ = 5.8 × 10^3^, 8.1 × 10^4^ s^–1^). This interesting observation has prompted us to reconsider the case of [Gd(AAZTA)(H_2_O)_2_]^−^ and, using new NMR relaxometric data obtained with a much more intense magnetic field (11.7 instead of 2.1 T), to verify if there is any experimental evidence that the two water molecules can be characterized by different water exchange rates.

The topic that we address in this work is of interest both for the area of MRI biomedical imaging and, above all, for basic coordination chemistry as it considers fundamental aspects of the properties of lanthanide complexes.

## Results and Discussion

### Relaxometric Characterization of [Gd(AAZTA)(H_2_O)_2_]^−^

**Figure 1:**
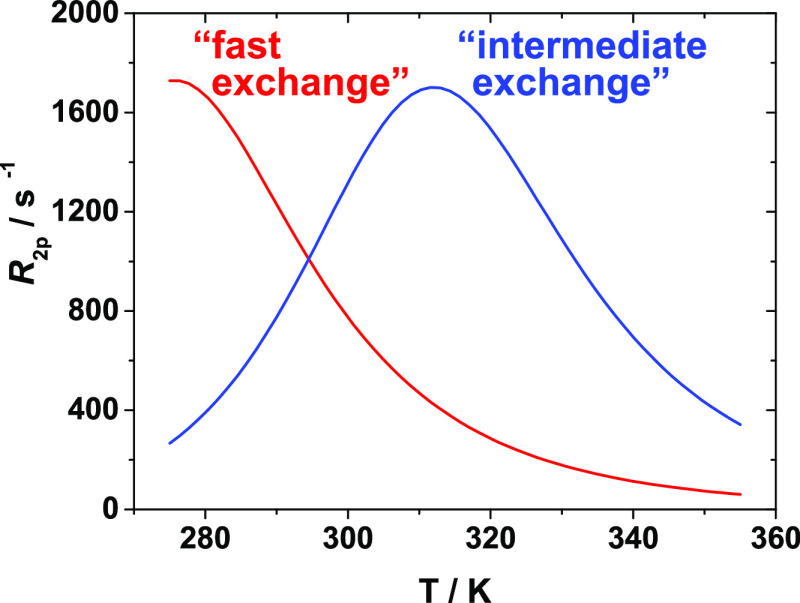
Temperature dependence of the ^17^O transverse relaxation rate calculated at 11.74 T for a monohydrated Gd(III)-complex 25 mM with a τ_M_ = 30 ns, Δ*H*^#^ = 40 kJ mol^–1^ (red profile) and a τ_M_ = 300 ns, Δ*H*^#^ = 48 kJ mol^–1^ (blue profile).^22^

NMR spectroscopy is able to provide an accurate description of the water exchange process that occurs between the paramagnetic center and the bulk solvent. Exchange rates of the metal-bound water molecules (*k*_ex_) can be obtained via high-resolution NMR, by measuring the paramagnetic shift (Δω) and the linewidths of the ^17^O signal of the isotopically enriched bulk water (Δν_1/2_(^17^O)), and hence its transverse relaxation rate (*R*_2_ = π*Δν_1/2_(^17^O)), as a function of temperature. The so-obtained data can be analyzed with the Swift–Connick equations that describe the water exchange process between the two sites.^21,22^ As a first approximation, the transverse relaxation rate can be considered inversely proportional to the sum of the residence lifetime of the water molecule coordinated to the metal center (τ_M_) and its transverse relaxation time (*R*_2_ ∝ 1/(*T*_2M_ + τ_M_)). On this basis, low-molecular-weight Gd(III)-chelates, characterized by τ_M_ values similar to *T*_2M_, at physiological temperatures, are predicted to follow an “intermediate exchange regime”, as reported for the [Gd(DTPA)(H_2_O)]^2–^ and [Gd(DOTA)(H_2_O)]^−^ complexes with a τ_M_ of about 300 ns at 298 K (Figure 1).^23^ Such chelates are expected to undergo a slow-to-fast exchange transition in the temperature range between 280 and 350 K. Indeed, at low temperatures (*T* = 280 K), a decrease of the water exchange rate and a shortening of *T*_2M_ occurs, which renders τ_M_ longer than *T*_2M_, thus the dominant term in the denominator of the *R*_2_ equation. In such exchange dynamics, described as the “slow-exchange regime”, the *R*_2_ values follow the same temperature dependence trend as τ_M_.^2^*R*_2_ is predicted to increase at rising temperatures (280 K < *T* < 310 K) to reach a maximum at 310 K, coinciding with the slow-to-fast exchange transition point. A further temperature increase (*T* > 310 K) induces an increase of the water exchange rate and an increase of *T*_2M_ (τ_M_ ≪ *T*_2M_), which is described as the “fast-exchange regime”. Now *T*_2M_ becomes predominant and dictates the behavior of the *R*_2_ temperature dependence, which decreases for increasing temperatures (Figure 1).

**Figure 2:**
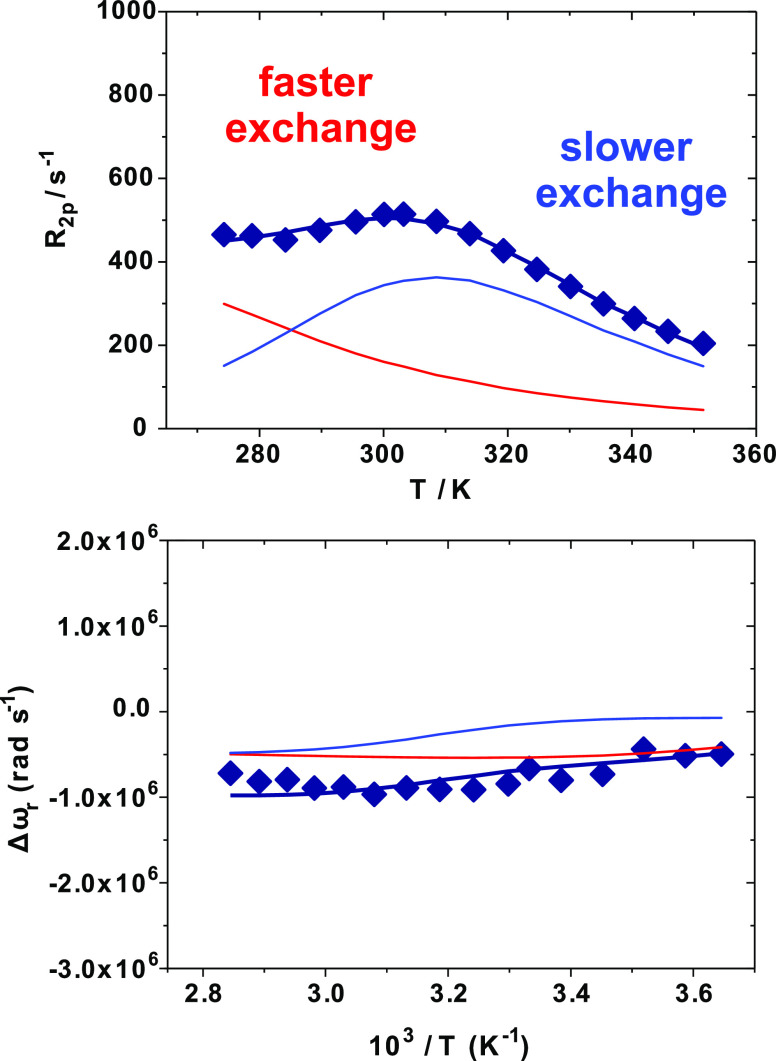
^17^O transverse relaxation rates (top) and ^17^O chemical shift variation (bottom) as a function of temperature measured for the [Gd(AAZTA)(H_2_O)_2_]^−^ complex 9.2 mM, pH 7.0, at 11.74 T. The solid lines correspond to the fits of the data as described in the text, while the red and blue lines represent the calculated contributions of the two different inner-sphere waters, in faster and slower exchange with the bulk, respectively.

While the temperature dependence of the ^17^O transverse relaxation rates previously measured on [Gd(AAZTA)(H_2_O)_2_]^−^ at a low magnetic field strength (*B*_0_ = 2.1 T) follows the expected behavior,^15^ the same measurements performed at higher magnetic fields (11.74 T) display an unusual trend in the lower temperature region (Figure 2). The increase in transverse relaxation rates at higher magnetic field strengths results in broader ^17^O NMR lines, measurable with better accuracy than at 2.1 T (Figure S1). This unraveled the presence of an unexpected trend in the relaxation profile at lower temperatures. Notably, an increase of *R*_2_ becomes clearly observable for temperature values lower than 285 K (Figure 2). Such a trend resembles that previously observed for [Gd(HPDO3A)(H_2_O)], characterized by the presence of multiple isomers in slow exchange on the NMR timescale, each featuring different water exchange rates.^24^ Instead, in the case of [Ln(AAZTA)(H_2_O)*_x_*]^−^ complexes, the existence of slow-exchange isomers is discarded by experimental evidence obtained from variable-temperature high-resolution NMR, where only one set of resonances is observable in the temperature range between 273 and 310 K.^19^

More likely, the behavior here reported could be owed to the presence of two Gd-coordinated water molecules with significantly different water exchange rates (*k*_ex_ = 1/τ_M_). Such a hypothesis is in agreement with recent studies, in which the presence of two metal-bound water molecules with significantly different residence times were suggested by means of density functional theory (DFT) calculations and wave function analyses.^25^ In this case, the two Gd(III)-coordinated water molecules are expected to contribute to a different extent to the observed ^17^O-*R*_2_ profile: the intermediate-exchanging species mainly contributes at intermediate temperatures, where the contribution of the faster-exchanging water molecule is almost absent. Conversely, the latter becomes predominant for *T* lower than ∼280 K, where the contribution of the slower-exchanging water is negligible. Previously, it was not possible to detect the presence of the faster exchange species at lower field strengths (*B*_0_ = 2.1 T).^15^

**Figure 3:**
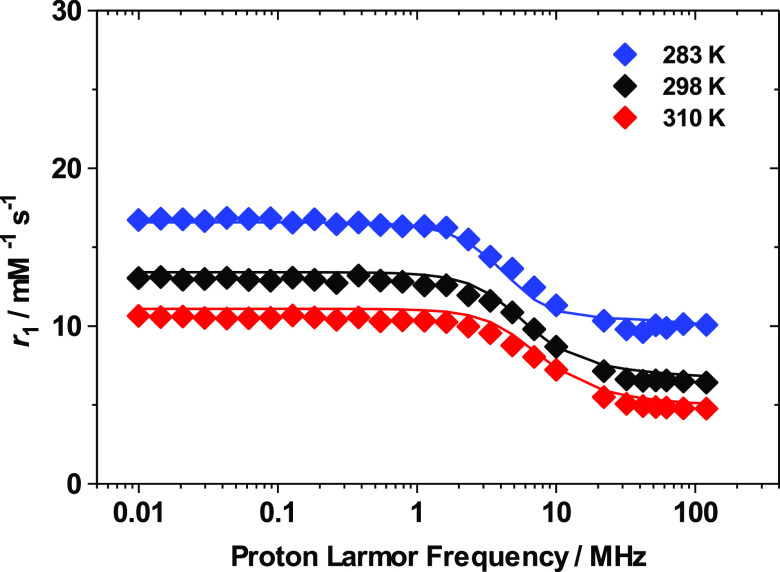
^1^H NMRD profiles of a 3.1 mM aqueous solution of [Gd(AAZTA)(H_2_O)_2_]^−^, at pH 7.0, recorded at different temperatures (283 K (solid blue diamonds), 298 K (solid black diamonds), and 310 K (solid red diamonds)). The solid lines correspond to the fits of the data, as described in the text.

On this basis, the ^17^O-*R*_2_ data were reanalyzed by considering the presence in solution of two water molecules subject to different dynamics of exchange. To obtain a comprehensive description of such processes, a complete set of high-field ^17^O NMR measurements was collected, comprising water ^17^O*-R*_2_ and chemical shift variations as a function of temperature (Figure 2), along with ^1^H nuclear magnetic relaxation dispersion (NMRD) profiles at three different temperatures (10, 25, and 37 °C) over the ^1^H Larmor frequency range 0.01 to 120 MHz (Figure 3). The ^1^H and ^17^O NMR data were simultaneously fitted to obtain more reliable results;^26^ the best-fit parameters are reported in Table 1. The Solomon–Bloembergen–Morgan and Freed equations^27−31^ were used for analyzing the ^1^H NMRD profiles, and in particular the inner-sphere and outer-sphere contributions to the relaxation, whereas the Swift–Connick equations were used to fit the ^17^O experimental data.^21^

**Table 1:** Parameters Obtained from the Fits of ^17^O NMR and ^1^H NMRD Data

	[Gd(AAZTA)(H_2_O)_2_]^−^	[Gd(AAZTA)(H_2_O)F]^2–^
*r*_1_^298^/mM^–1^ s^–1^ (32 MHz)	6.6	4.2
^298^Δ^2^/10^19^ s^–2^	2.6	2.7
^298^τ_V_/ps	30	21
*E*_V_/kJ mol^–1^	1.0^a^	1.0^a^
*A*_O_^A^/ℏ/10^6^ rad s^–1^	–3.8	
*A*_O_^B^/ℏ/10^6^ rad s^–1^	–3.9	–3.8
^298^τ_M_^A^/ns	29	
Δ*H*_M_^A^/kJ mol^–1^	20.0	
^298^τ_M_^B^/ns	169	3.4
Δ*H*_M_^B^/kJ mol^–1^	29.5	23.4
^298^τ_R_/ps	74.0	74.0
*E*_R_/kJ mol^–1^	20.0	24.0
*C*_os_	0.02	0.0
*q*	2^a^	1^a^
*r*/Å	3.05^a^	3.05^a^
*a*/Å	4.0^a^	4.0^a^
^298^*D*/10^5^ cm^2^ s^–1^	2.24^a^	2.24^a^
*E*_D_/kJ mol^–1^	20.0^a^	20.0^a^

a Parameters fixed during the fitting procedure.

Some of the parameters affecting *r*_1_ and the ^17^O NMR data were fixed to the values reported in the literature for [Gd(AAZTA)(H_2_O)_2_]^−^ and its derivatives:^15^ the number of water molecules coordinated to the metal ion (*q* = 2), the distance between the inner-sphere water protons and Gd(III) (*r* = 3.05 Å), the closest distance between an outer-sphere water molecule and the paramagnetic center (*a* = 4.0 Å), the relative diffusion coefficient of outer-sphere water molecules and the complex at 298 K (^298^*D* = 2.24 × 10^5^ cm^2^ s^–1^), and the activation energy for the diffusion coefficient (*E*_D_ = 20 kJ mol^–1^).

The data were well reproduced by considering that the two Gd(III)-bound water molecules are characterized by significantly different residence lifetimes (τ_M_^A^ = 29 ns and τ_M_^B^ = 169 ns) and enthalpy barriers associated with the exchange process (Δ*H*_M_^A^ = 20 kJ mol^–1^ and Δ*H*_M_^B^ = 29.5 kJ mol^–1^). It is worth noting that the average of these two residence lifetimes is very similar to that obtained previously under the assumption that the two coordinated water molecules are characterized by identical water exchange dynamics (τ_M_ = 90 ns).^15^ The water molecule residing for shorter times on the metal center (A) exchanges ∼6 times faster than the other, with a reaction enthalpy ∼1.5 times lower. This suggests that the exchange reaction is mainly controlled by the energy cost needed to break one Gd–Ow bond, necessary to reach an eight-coordination transition state. It is reasonable to assume that breaking the Gd–Ow bond of the water molecule that is less strongly bound to the metal center, hence at a longer distance from it, requires the lowest energy. This is in line with previous predictions of Platas-Iglesias and co-workers, who calculated significantly different Gd–Ow distances for the two water molecules (*r*^A^ = 2.505 Å and *r*^B^ = 2.484 Å) differently positioned in the Gd(III)-coordination environment. The considerably more labile water molecule occupies a capping position, and the more tightly bound to the metal center resides in one of the vertices of the coordination polyhedron.^20^ This is in agreement with the labile capping bond effect introduced previously, which states that water ligands occupying sterically demanding capping positions are intrinsically labile.^32^ The calculated ^17^O hyperfine coupling constants show a slightly higher value for the more strongly bound and more slowly exchanging water molecule (*A*_O_/ℏ^A^ = −3.8 × 10^6^ rad s^–1^ and *A*_O_/ℏ^B^ = −3.9 × 10^6^ rad s^–1^), consistent with the DFT predictions.^25^ The rotational dynamics (τ_R_ = 74.0 ps) and electronic parameters (Δ^2^ = 2.6 × 10^19^ s^–2^; τ_V_ = 30 ps) characterizing the system are in agreement with previous studies reported in the literature.^15^

The influence of the simultaneous presence of the two species is not evident in the dependence of the relaxivity (*r*_1_) on the magnetic field strengths (Figure 3), as proton relaxivity is limited by the fast rotation of the complex in solution rather than by water exchange.

### Affinity Constant of the Fluoride Anion for the [Gd(AAZTA)(H_2_O)_2_]^−^ Complex

Previous studies demonstrated that fluoride can directly compete with water molecules to coordinate lanthanide complexes and remain tightly bound.^33−35^ The halide affinity for the metal center can be sufficiently high to replace one or more inner-sphere water molecules.^9^ As a result, the paramagnetic metal ion loses efficiency in relaxing the bulk water protons, which leads to a significant decrease in relaxivity, conveniently detectable by relaxometry.

**Figure 4:**
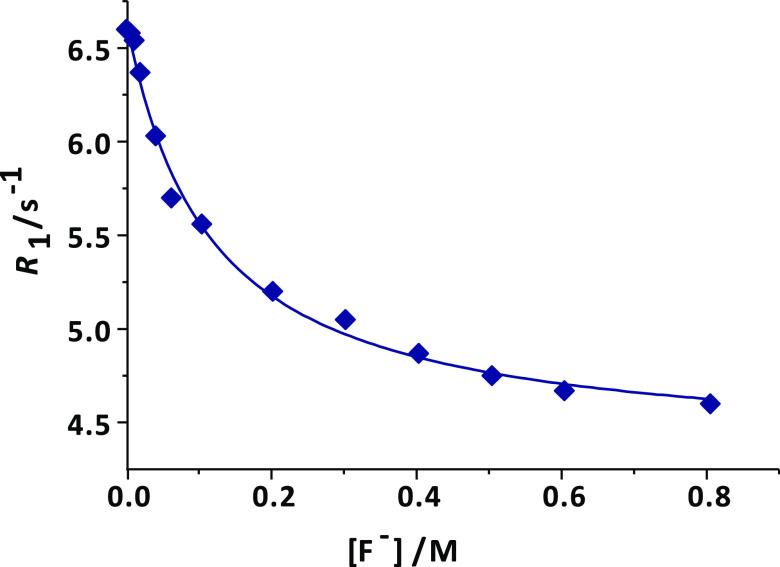
Relaxometric titration of 1 mM aqueous solution of [Gd(AAZTA)(H_2_O)_2_]^−^ with increasing amounts of NaF (32 MHz and 298 K). The solid lines correspond to the fits of the data, as described in the text.

We investigated the binding of the fluoride anion to [Gd(AAZTA)(H_2_O)_2_]^−^ by monitoring the change in the longitudinal relaxation rates of water protons (*R*_1_), which occurs after adding increasing amounts of the halide to the aqueous solution of the complex. For this purpose, a 1 mM [Gd(AAZTA)(H_2_O)_2_]^−^ solution was titrated with NaF until variations of *R*_1_ become negligible, which corresponds to an 800-fold excess of the halide. Changes of *R*_1_ were measured at 32 MHz, 298, and 310 K (Figures 4 and S2). The so-obtained titration curves were analyzed following the proton relaxation enhancement (PRE) method, which provides access to the apparent affinity constant *K*_a_ and to the relaxivity of the ternary complex, *r*_1bound_ (Table 2).^2^ At *B*_0_ = 32 MHz and 298 K, the [Gd(AAZTA)(H_2_O)F]^2–^ adduct shows a relaxivity value lower than that of the corresponding binary complex, and compatible with the loss of only one of the coordinated water molecules (^298^*r*_1bound_ = 4.2 mM^–1^ s^–1^). Thus, the replacement of the second inner-sphere water molecule by the fluoride anion appears to be unfavorable due to the high electrostatic repulsion occurring among the two anions.

**Table 2:** Refinement Parameters for the Binding of NaF to the [Gd(AAZTA)(H_2_O)_2_]^−^ Complex Measured at 298 and 310 K

	298 K	310 K
[Gd(AAZTA)(H_2_O)_2_]^−^ (mM)^a^	1.0	1.1
^32MHz^*r*_1free_ (mM^–1^ s^–1^)^a^	6.6	5.0
*r*_1bound_ (mM^–1^ s^–1^)	4.2 ± 0.06	3.0 ± 0.06
*q*_bound_^a^	1	1
χ^2^	0.004	0.005
*K*_a_ (M^–1^)	8.8 ± 0.8	8.0 ± 0.7

a Parameters fixed during the fitting procedure.

**Scheme 2:**
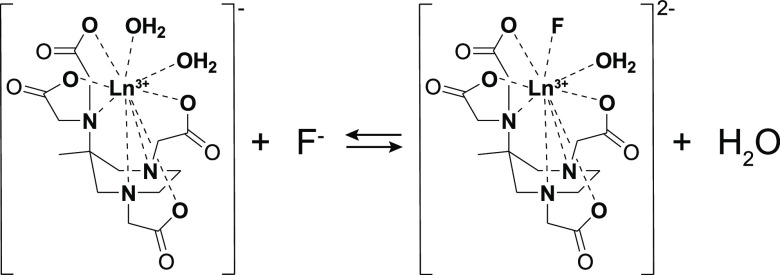
Equilibrium between the Hydrated and Fluoride-Bound Forms of the Ln(AAZTA) Complexes under Study

The low values of the affinity constants (*K*_a_ = 8.8 and 8.0 M^–1^ at 298 and 310 K, respectively) are attributable to the weak interaction of the anion with the [Gd(AAZTA)(H_2_O)_2_]^−^ complex, caused by the strong electrostatic repulsion between the fluoride anion and the negatively charged complex. Similar affinity constants were determined for the binding of F^–^ to the bis-hydrated negatively charged [Gd(DTTA-Me)(H_2_O)_2_]^−^ complex. At the end of the titration, a mixture of the binary and ternary complexes is present in solution, where the concentration of the binary adduct is estimated to be 12% of the total (Scheme 2). As a consequence, the relaxometric properties of the so-prepared solution arise from two different contributions: (1) one related to the preponderant ternary species having only one inner-sphere water molecule, which contributes to a minor extent to the observed relativity, and (2) the dominant contribution of the minor binary species with two coordinated water molecules. To characterize the relaxometric properties of the pure [Gd(AAZTA)(H_2_O)F]^2–^, the complete formation of the ternary complex should be obtained, which can be only achieved using extremely high concentrations of the halide (≥1 M). However, it is known that high ionic strengths (>0.6 M) can substantially alter the relaxometric properties of the chelates in aqueous solutions, due to the strong salt-water interactions capable of modifying the structure and microviscosity of water and therefore, of changing the molecular reorientation rates of the complexes.^36^

Therefore, to investigate the relaxometric behavior of the [Gd(AAZTA)(H_2_O)F]^2–^ complex, 0.6 M NaF was added to the solution of [Gd(AAZTA)(H_2_O)_2_]^−^, and the relaxometric contribution of the binary species, calculated on the basis of its molar fraction present in solution for each working temperature, was subtracted from the total, as explained below.

### Relaxometric Characterization of the [Gd(AAZTA)(H_2_O)F]^2–^ Ternary Complex

**Figure 5:**
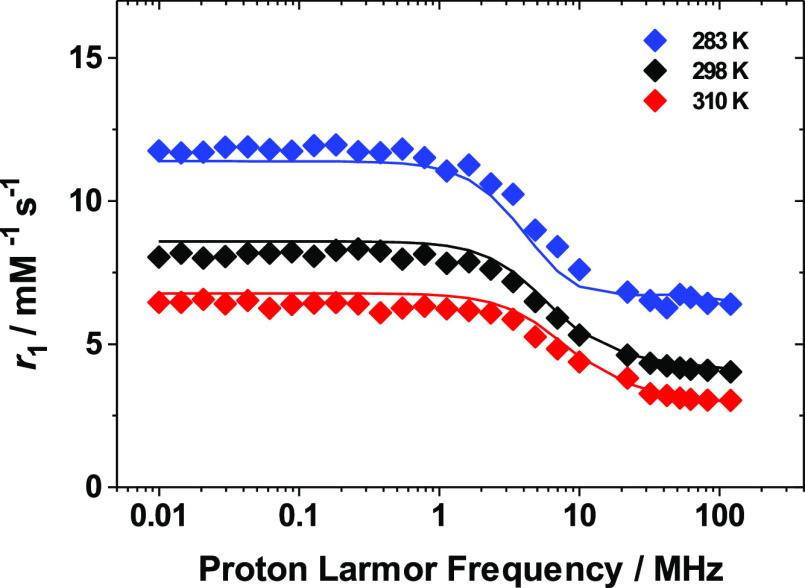
^1^H NMRD profiles of a 1.0 mM solution of [Gd(AAZTA)(H_2_O)F]^2–^, in the presence of NaF 600 mM, recorded at different temperatures 283 K (solid blue diamonds), 298 K (solid black diamonds), and 310 K (solid red diamonds). The contribution to the relaxivity of the residual [Gd(AAZTA)(H_2_O)_2_]^−^ species was subtracted from the NMRD profiles of [Gd(AAZTA)(H_2_O)F]^2–^ for each temperature, as described in the text. The solid lines correspond to the fits of the data.

**Figure 6:**
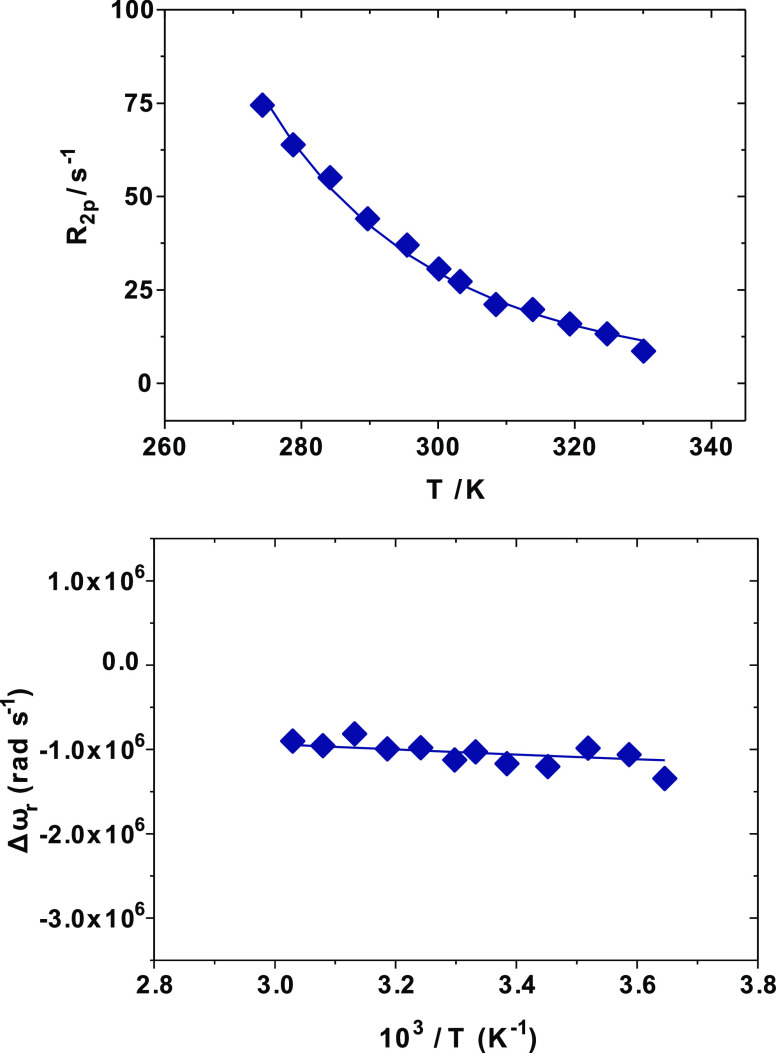
^17^O transverse relaxation rates (top) and ^17^O chemical shift variation (bottom) as a function of the temperature of [Gd(AAZTA)(H_2_O)F]^2–^ 9.4 mM, in the presence of NaF 600 mM, measured at 11.74 T. The contribution to the relaxivity of the residual [Gd(AAZTA)(H_2_O)_2_]^−^ species was subtracted from the ^17^O profiles of [Gd(AAZTA)(H_2_O)F]^2–^ for each temperature, as described in the text. The solid lines correspond to the fits of the data.

Relaxometric characterization of the ternary adduct, in which one water molecule is replaced with a fluoride anion, was performed with the aim of evaluating whether any changes in water exchange dynamics were detectable. To characterize the relaxometric properties of the ternary complex alone, a complete set of ^1^H NMRD profiles and variable-temperature ^17^O NMR data was acquired on a [Gd(AAZTA)(H_2_O)F]^2–^ solution, from which the binary adduct contribution to the relaxation was subtracted for each of the temperatures measured (Figures 5 and 6).

As already mentioned, the ternary complex was prepared by adding 0.6 M of NaF to a 1 mM [Gd(AAZTA)(H_2_O)_2_]^−^ solution. The concentration of the binary adduct present in such a solution was calculated for all of the measuring temperatures from the enthalpy of the fluoride–Gd-binding reaction, which was derived from the *K*_a_ values reported in Table 2, assuming an Arrhenius behavior. Then, a solution of the binary complex having the same ionic strength as the ternary species was prepared by adding 0.6 M NaCl to a 1 mM solution of [Gd(AAZTA)(H_2_O)_2_]^−^. The same set of ^1^H and ^17^O NMR data were acquired on the two samples under exactly the same conditions. There is clear evidence that the addition of NaCl to the complex does not affect the number of inner-sphere water molecules and their dynamic properties (Figure S3 and Table S1). This allowed subtracting, for each measurement, the contribution to the relaxation of the binary complex from that of the ternary species, to obtain the relaxation profiles of the monohydrated complex alone, free from possible influences of the relatively high ionic strength. The simultaneous analysis of the magnetic field dependence of *R*_1_ of the water protons, and of the temperature dependence of *R*_2_ and Δω of ^17^O (Figures 5 and 6), was performed using the same approach described above, to obtain quantitative information on the exchange dynamics of the monohydrated complex. During the fitting procedure, the same set of parameters described above was fixed to the values used for the binary [Gd(AAZTA)(H_2_O)_2_]^−^ complex (Table 1), except for the hydration number that was set to 1.

By reasonably assuming that F^–^ more easily substitutes the more labile water molecule, characterized by a shorter residence lifetime (τ_M_^A^ = 29 ns), the longer bound distance from the metal center (*r* = 2.505 Å),^20,25^ and lower energy cost associated with breaking of the Gd–Ow bond (Δ*H*_M_^A^ = 20 kJ mol^–1^) and that its binding reduces the charge density at the metal center and labilizes the coordinated water molecule, we expect an acceleration of the water exchange rate. This is in agreement with the experimental data showing a remarkable (∼50-fold) decrease in the residence lifetime (τ_M_ = 3.4 ns), followed by a slight reduction of the enthalpy associated with the exchange process (Δ*H*_M_ = 23.4 kJ mol^–1^) with respect to the more strongly bound water molecule of the binary complex. This behavior may be favored by the increase in the negative charge of the complex and by the steric interaction in the water coordination site that could destabilize the nine-coordinate ground state, thus diminishing the activation energy for the exchange process. However, the value of the hyperfine coupling constants (*A*_O_/ℏ = −3.8 × 10^6^ rad s^–1^) remains virtually unaffected.

DFT calculations were performed to rationalize the results of the relaxometric study (Tables S3–S5). The model systems investigated include a number of explicit second-sphere water molecules, while bulk solvent effects were considered using a polarized continuum model. Different computational studies evidenced that this mixed cluster/continuum approach is required to attain a proper description of the Ln–Ow and Ln–F bonds.^37,38^

**Figure 7:**
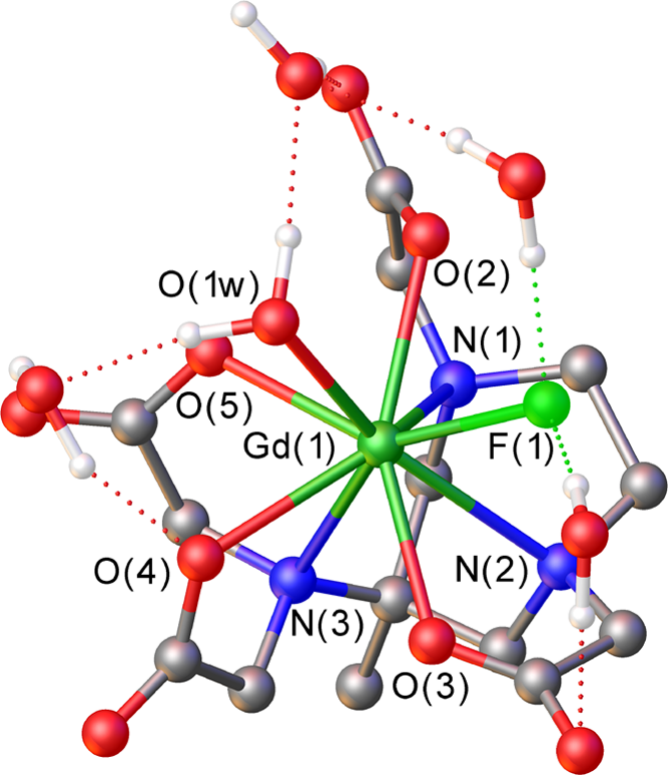
Structure of the [Gd(AAZTA)(H_2_O)F]^2–^ system obtained using DFT calculations. Selected bond distances (Å): Gd(1)–F(1), 2.271; Gd(1)–N(1), 2.718; Gd(1)–N(2), 2.713; Gd(1)–N(3), 2.628; Gd(1)–O(1w), 2.495; Gd(1)–O(2), 2.414; Gd(1)–O(3), 2.445; Gd(1)–O(4), 2.456; and Gd(1)–O(5), 2.431.

Calculations performed on the [Gd(AAZTA)(H_2_O)F]^2–^·5H_2_O system suggest that fluoride coordination may replace preferentially the water molecule that provides the weakest interaction with the metal ion (Figure 7), as the free energy difference between the two ternary adducts is large (∼4.6 kcal mol^–1^) (Tables S3 and S4). Fluoride coordination induces a significant lengthening of the Gd–Ow distance from 2.480 Å in [Gd(AAZTA)(H_2_O)_2_]^–^ to 2.495 Å in [Gd(AAZTA)(H_2_O)F]^2–^·5H_2_O, using the same computational approach. This is in agreement with the acceleration of the water exchange observed experimentally. Thus, fluoride coordination appears to weaken the interaction between the remaining coordinated water molecule and the metal ion, thus facilitating water exchange following a dissociative mechanism. This is confirmed by the electron density calculated at the (3,–1) critical point^39^ characterizing the Gd–Ow bond (ρ_BCP_), which decreases from 3.93 × 10^–3^ to 3.74 × 10^–3^ au upon fluoride coordination. Different computational studies on lanthanide complexes showed that lower ρ_BCP_ values characterize weaker Ln–Ow bonds.^40,41^ The calculated Laplacian bond orders (LBOs)^42^ also point to weaker coordination of the water molecule in [Gd(AAZTA)(H_2_O)F]^2–^·5H_2_O (LBO = 0.100) than in [Gd(AAZTA)(H_2_O)_2_]^−^·4H_2_O (LBO = 0.125).

### Kinetics and Thermodynamics of the Fluoride Binding with [Y(AAZTA)(H_2_O)_2_]^−^

The presence of an NMR active ^19^F species transiently coordinated to the paramagnetic center in the ternary complex prompted us to study its binding interaction by high-resolution NMR lineshape analysis. However, the long electronic relaxation time that characterizes Gd(III) (τ_S_ ∼ 10^–8^ s) gives the paramagnetic metal ion an excellent relaxing capability, which causes severe broadening of the NMR lines of the neighboring nuclei. To access the ^19^F resonances necessary to evaluate the exchange rate between the metal-bound and free fluoride, we substituted Gd(III) with the diamagnetic analogue Y(III). Despite the structure of the Ln(III)AAZTA complexes varying as a function of the lanthanide contraction, the rather similar ionic radii of Gd(III) and Y(III) are expected to preserve the coordination geometry and the water dynamics properties.^43^ However, we cannot exclude the presence in solution of two species with different hydration numbers (*q* = 1, 2) in fast exchange. By means of variable-temperature high-resolution ^19^F NMR measurements, it was possible to access the kinetic information on the exchange rate of the chemical reaction, and therefore the thermodynamic parameters associated with fluoride exchange.

A quantitative evaluation of the chemical exchange rate was obtained on a sample containing a mixture of the fluoride-free and fluoride-bound species that are characterized by distinct ^19^F resonances, by monitoring variations of the line shapes induced upon temperature changes.

**Figure 8:**
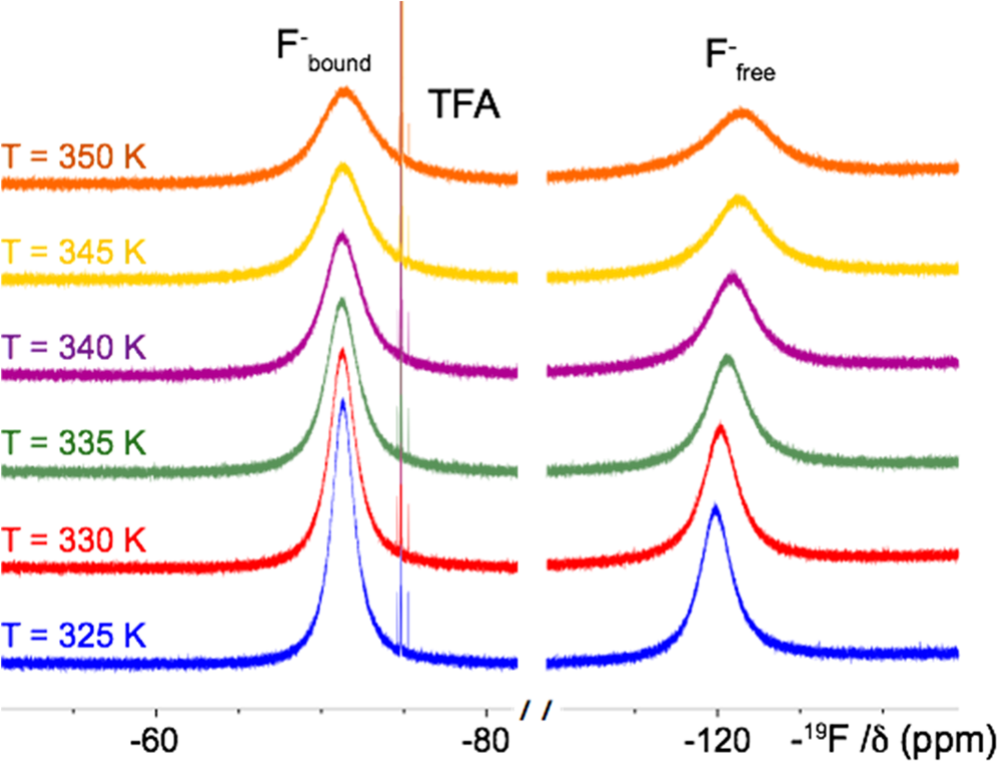
One-dimensional ^19^F NMR spectra acquired on the [Y(AAZTA)(H_2_O)F]^2–^ ternary complex. The adduct was prepared by adding NaF (260 mM) to a solution of [Y(AAZTA)(H_2_O)_2_]^−^ (160 mM). The spectra were acquired at 11.74 T in the temperature range from 325 to 350 K. Three signals resonating at −72, −75, and −120 ppm correspond to bound fluoride, trifluoroacetate, and free fluoride, respectively.

The sample was prepared to ensure a ∼1 to 1 molar ratio of fluoride in the free (F_free_^–^) and bound (F_bound_^–^ = [Y(AAZTA)(H_2_O)F]^2–^) form at the lowest working temperature, to obtain a system with a symmetrical two-site exchange reaction. This facilitates measurements of the linewidths and therefore determination of the fluoride exchange rate (see Experimental Section). High-resolution one-dimensional (1D) ^19^F NMR spectra were acquired by varying the temperature between 275 and 350 K. The simultaneous presence of two distinct sets of signals associated with the F_bound_^–^ (−72 ppm) and F_free_^–^ (−120 ppm) species indicates that the exchange between the free and bound species is slow on the NMR timescale (Figure 8). The third signal resonating at −75 ppm was assigned to the trifluoroacetate (TFA) present in the solution of the complex after deprotection.

It is worth noting that DFT calculations performed on the [Y(AAZTA)(H_2_O)F]^2–^ system provide a calculated ^19^F NMR shift of −82.7 ppm, in reasonable agreement with the experimental value of −72 ppm. Calculations on the F^–^·18H_2_O system give a calculated ^19^F chemical shift of −125.3 ppm, in excellent agreement with the experiment. These results clearly indicate that DFT provides a reasonable model for the structure of the fluoride adduct in solution.

The integration of the two peaks allows calculating the relative concentration of the F_bound_^–^ and F_free_^–^ species for all of the measured temperatures. Significant variations of the linewidths of the fluoride-free and bound forms were detected only above 325 K (Figure S4), suggesting that the fluoride exchange rate increases at increasing temperatures, and tends to reach the coalescence point for temperatures higher than 350 K (Figure 8). The fluoride exchange rate was determined by fitting the ^19^F integrals and linewidths at various temperatures, with respect to the TFA reference signal, through the dynamic NMR (DNMR) LineShape Analysis tool (version 1.1.2) implemented in Bruker’s Topspin 3.2 (Figure S4). The fluoride exchange rate is 2280 s^–1^ at 325 K (Table S2), 1 order of magnitude faster than that found for a similar system, a tripositive Y(III)DOTA-tetraamide derivative at 298 K.^34^ This difference in rates is attributable to the electrostatic repulsion occurring between the fluoride anion and the metal center in the negatively charged [Y(AAZTA)(H_2_O)_2_]^−^ complex, which significantly accelerates the dissociation reaction with respect to the tripositive complex.

**Figure 9:**
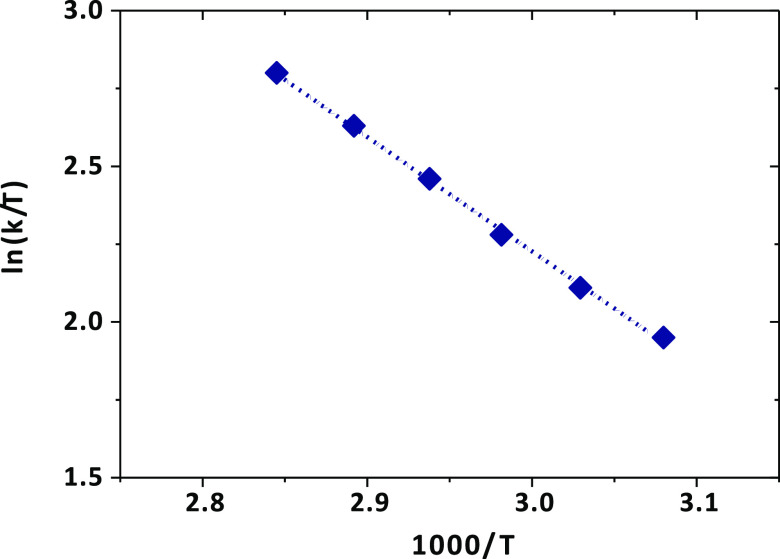
Eyring plots for the exchange rates of the fluoride-free and [Y(AAZTA)(H_2_O)_2_]^−^-bound form. Dashed lines represent the fitted data.

The plot of the logarithm of the kinetic constant as a function of temperature provides a linear correlation that allows estimating the thermodynamic parameters of the exchange reaction. The enthalpy (Δ*H*^#^) and entropy (Δ*S*^#^) variations of the reaction are extracted from the slope and intercept of the Eyring plot (Figure 9) and enable determining the variation of the Gibbs free energy (Δ*G*^#^) associated with the exchange process (Table S2).

The enthalpy difference extracted from the plot (Δ*H*^#^ = 32.26 kJ mol^–1^) is lower than that reported for other tripositive Y(III)DOTA-tetraamide derivatives (Δ*H*^#^ = 45–47 kJ mol^–1^), suggesting that the energy cost for the bond breaking between the fluoride anion and the metal center in a negatively charged complex is lower than that of a tripositive chelate.

Conversely, the entropy variation (Δ*S*^#^ = −82.2 J mol^–1^ K^–1^) is significantly different from that reported for Y(III)DOTA-tetraamide derivatives (Δ*S*^#^ = −41 to 58 J mol^–1^ K^–1^) indicating that the fluoride–metal ion interaction is appreciably affected by the nature of the ligand substituents, as well as the structure and the charge of the complex.

## Conclusions

The complex [Gd(AAZTA)(H_2_O)_2_]^−^ represents one of the few bis-aquated Gd^3+^ derivatives that displays favorable properties for *in vivo* applications as an MRI contrast agent. Bis-hydrated Gd(III)-complexes have been developed with the aim of improving the efficacy of GBCAs, which in principle may allow the injection of lower Gd^3+^ doses in clinical practice and biochemical research. Only recently, unpredictable and unique coordination properties have been proven to characterize Ln(III)-complexes of AAZTA, which have not yet been fully characterized and understood. These concern the decrease of the hydration state along the lanthanide series due to the lanthanide contraction, which is unexpectedly associated with a significant decrease of the exchange rate of the remaining inner-sphere water molecule. This behavior is similar to that shown by the eight-coordinate [Ln(PDTA)(H_2_O)_2_]^−^ derivatives, but in contrast to that observed for the nine-coordinate [Ln(DTPA–BMA)(H_2_O)] and [Ln(DO3A)(H_2_O)_2_] complexes. The detailed NMR studies reported here, allowed us to reveal subtle details of the coordination chemistry of [Gd(AAZTA)(H_2_O)_2_]^−^ and to address one of the questions that remained unanswered, that is whether the two water molecules coordinated to the metal ion are characterized by different residence times in the metal coordination sphere. The new ^1^H NMRD and particularly ^17^O NMR data, collected at higher magnetic field strengths, provided clear evidence that the two identical water ligands have substantially different exchange rates, one being considerably more labile than the other.

Such finding is supported by DFT calculations predicting that the faster-exchanging water molecule is more distant from the metal center and occupies the sterically hindered capping position of the coordination polyhedron, while the slower-exchanging water, located at one of the vertices of the coordination polyhedron, is closer to the metal center.

In addition, it has been shown that the more labile water molecule can be replaced by fluoride to form a [Gd(AAZTA)(H_2_O)F]^2−^ adduct, which is characterized by a fast water exchange rate (τ_M_ = 3.4 ns at 298 K). Fluoride exchange is conversely very slow and could be analyzed by high-resolution NMR using the diamagnetic Y(III) derivative (τ_M_ = 1.4 ms at 298 K).

The resulting picture clarifies the mechanism by which the increased steric compression induced by the contraction of the ionic radius along the Ln series favors the loss of the more labile water molecule. As a result, the coordinated water molecule with a lower steric hindrance interacts more tightly and resides for longer times at the metal center.

**Figure 10:**
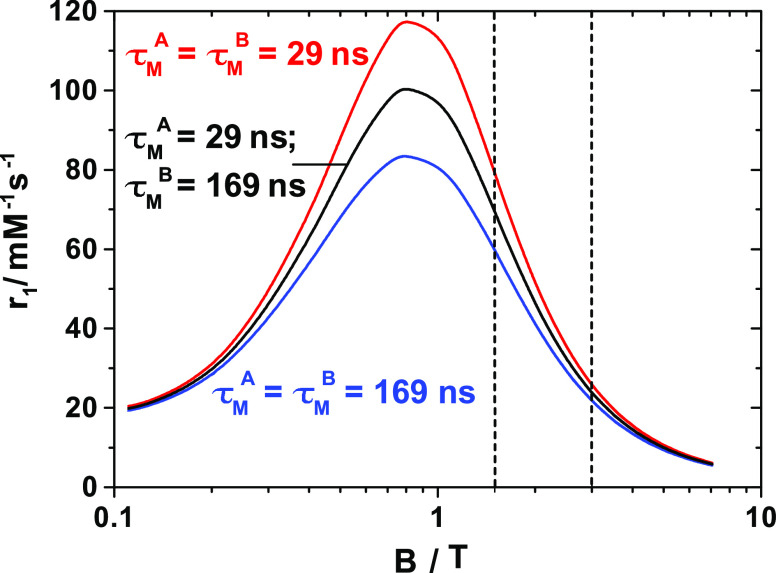
Relaxivity values calculated for a bis-hydrated Gd(III) complex with a τ_R_ = 5 ns and different water residence times. All other parameters are those listed for [Gd(AAZTA)(H_2_O)_2_]^−^ in Table 1. Vertical dashed lines indicate magnetic fields of 1.5 and 3 T.

These results, in addition to their relevance to the coordination chemistry of f-elements, also indicate an approach to improve the effectiveness of this complex as an MRI probe for clinical and preclinical applications. One of the possible avenues to take could be inducing structural changes of the coordination geometry to drive the selective acceleration of the slow-exchanging water molecule while keeping the other one in an optimal exchange regime. This would induce significant relaxivity gains if the rotational dynamics is slowed down, for instance by interaction with human serum albumin (HSA). It is worth noting that lipophilic GdAAZTA derivatives retain the two coordinated water molecules when bound to HSA. In fact, theoretical predictions estimate an increase in *r*_1_ for macromolecular GdAAZTA derivatives (τ_R_ = 5 ns) at the clinically relevant magnetic fields of 1.5 and 3 T (Figure 10). At 1.5 T, these simulations predict a relaxivity gain of ∼14% (from *r*_1_ = 69.5 to 79.2 mM^–1^ s^–1^) by reducing the residence time of the water molecule with τ_M_ = 169 ns to 29 ns. Acceleration of water exchange results in an *r*_1_ increase of ∼33% when taking as a reference a system in which both water molecules are characterized by τ_M_ = 169 ns (*r*_1_ = 59.7 mM^–1^ s^–1^). The effect is less pronounced at 3 T assuming τ_R_ = 5 ns but more important for an intermediate τ_R_ of 0.5 ns (Figure S5). Therefore, in light of these new findings, we envisage that the relaxation properties of GdAAZTA derivatives can be optimized by modifying the ligand structure, for example, by replacing one or two carboxylate moieties with more sterically demanding donor groups (as propionate or phosphonate arms).^44^

Taken together, the results reported in this paper represent a step forward toward an understanding of the structural and dynamic properties of lanthanide complexes with polyamino–polycarboxylate ligands, an important family of complexes because of their biomedical applications.

## Experimental Section

### NMR Experiments

^1^H, ^13^C, and NMR spectra of the ligands and their precursors were recorded at 298 K using a Bruker AVANCE III 500 spectrometer equipped with a 5 mm double resonance TXI probe.

### Preparation of the [Ln(AAZTA)(H_2_O)_2_]^−^ Complexes

The AAZTA ligand was kindly provided by Bracco Imaging S.p.A. (purity >99%).

[Ln(AAZTA)(H_2_O)_2_]^−^ complexes were prepared by adding 1.1 equiv of LnCl_3_ salts to an aqueous solution of the AAZTA ligand at pH = 6. After the addition, the pH was adjusted to 6.0 with dilute NaOH and the solution was stirred at room temperature (r.t.) for 12 h. Then, the pH was increased to 10 by means of 0.1 M NaOH, and the solution was stirred for 3 h, to promote the precipitation of the uncomplexed Ln(III) as insoluble hydroxides. The solution was centrifuged (10 000 rpm, 5 min, r.t.), the supernatant was filtered through 0.2 μm filters and neutralized with dilute HCl. The concentration of Ln(III) complexes was evaluated by ^1^H NMR measurements, using Evans’ method. The 1D ^1^H NMR spectrum of the diamagnetic [Y(AAZTA)(H_2_O)_2_]^−^ complex demonstrates the high purity of the complex (>95%) (Figure S6).

### Fluoride Affinities for the [Gd(AAZTA)(H_2_O)_2_]^−^ Complex

Association constants of [Gd(AAZTA)(H_2_O)F]^2−^ were determined by relaxometric titrations. The titrations were performed at 32 MHz, at two different temperatures (298 and 310 K), by adding increasing amounts of the NaF salt to 1 mM [Gd(AAZTA)(H_2_O)_2_]^−^ solution. Relaxivity values were measured after each addition for monitoring the formation of the ternary complex. A total of 13 points were collected for each titration. The final concentration of NaF was 0.8 M, corresponding to an 800-fold molar excess of the anion.

The same titration was performed in identical conditions, at 298 K, by adding increasing amounts of NaCl to a 1 mM [Gd(AAZTA)(H_2_O)_2_]^−^ solution.

NMRD profiles were acquired on the ternary [Gd(AAZTA)(H_2_O)F]^2−^ complex, prepared by adding 0.6 mM NaF salt to a 1 mM [Gd(AAZTA)(H_2_O)_2_]^−^ solution, and on the binary [Gd(AAZTA)(H_2_O)_2_]^−^ complex, prepared by adding 0.6 mM NaCl salt to a 1 mM [Gd(AAZTA)(H_2_O)_2_]^−^ solution (Figure S3a).

### Fluoride Affinity for the [Y(AAZTA)(H_2_O)_2_]^−^ Complex

The rate of fluoride exchange with [Y(AAZTA)(H_2_O)_2_]^−^ was determined by ^19^F high-resolution NMR measurements. The ternary complex was prepared by titrating a 160 mM [Y(AAZTA)(H_2_O)_2_]^−^ solution with increasing amounts of NaF at 275 K. After each addition, high-resolution 1D ^19^F spectra were acquired and the integrals of the ^19^F NMR signals of the fluoride-free and bound forms were measured to monitor the formation of the [Y(AAZTA)(H_2_O)F]^2–^ complex. At the end of the titration, an equal molar ratio of the fluoride-free and bound forms was reached, corresponding to a NaF concentration of 280 mM. 1D ^19^F spectra were acquired by varying the temperature from 275 to 350 K.

Delay times (D1) were set to 10 s to make integration quantitative. Spectra were phased and baselined before analysis. The rate of exchange of fluoride was calculated fitting the ^19^F line shapes of the F_free_ and F_bound_ species referred to the TFA peak, at various temperatures through the dynamic NMR (DNMR) LineShape Analysis module (version 1.1.2) implemented using Bruker’s Topspin 3.2, as detailed in Figure S4.

### Relaxometric Measurements

1/*T*_1_^1^H nuclear magnetic relaxation dispersion (NMRD) profiles were acquired using two different instruments, one operating at lower field strengths (0.01 to 10 MHz) and the other at higher field strengths (20–120 MHz). Low field data were measured using a fast-field cycling (FFC) Stelar SMARTracer relaxometer (Stelar s.r.l., Mede, PV, Italy) equipped with a silver magnet. High-field measurements were collected with a high-field relaxometer (Stelar) equipped with an HTS-110 3T Metrology cryogen-free superconducting magnet. The measurements were performed using the standard inversion recovery sequence (20 experiments and 2 scans) with a typical 90° pulse width of 3.5 μs and the reproducibility of the data was within ±0.5%. The temperature was controlled with a Stelar VTC-91 heater airflow equipped with a copper–constantan thermocouple (uncertainty of ±0.1 K).

### ^17^O Measurements

Variable-temperature ^17^O NMR measurements were recorded on a Bruker AVIII 500 spectrometer equipped with a 5 mm probe and standard temperature control unit. Solutions containing 2.0% of the ^17^O isotope (Cambridge Isotope) and 10% D_2_O for the external lock were used. The observed transverse relaxation rates were calculated from the signal full width at half-maximum (Δν_1/2_). The bulk magnetic susceptibility contribution was subtracted from the ^17^O NMR shift data using the ^1^H NMR shifts of the *t*BuOH signal as the internal reference. Other details of the instrumentation, experimental methods, and data analysis have been previously reported.^45^

### DFT Calculations

The structures of the gadolinium and yttrium complexes were optimized with density functional theory (DFT) calculations with the M062X exchange–correlation functional^46^ and the Gaussian16 software package.^47^ In these calculations, we used quasi-relativistic effective core potentials including 28 and 53 electrons in the core for Y (ECP28MWB) and Gd (ECP53MWB), together with their associated (8s7p6d2f1g)/[6s5p3d2f1g] (Y) and (7s6p5d)/[5s4p3d] (Gd) basis sets.^48,49^ The standard 6-311G(d,p) basis set was used for all other atoms (C, H, F, N, and O). Analytical second derivatives were performed to confirm the nature of the optimized geometries as local energy minima (0 imaginary frequencies). The integration grid was set with the integral = ultrafine keyword, while solvent effects were incorporated using a polarized continuum model (scrf = pcm, solvent = water).^50^ Wave function analysis was carried out with Multiwfn 3.2.^51^

^19^F NMR shielding tensors were calculated with the ORCA suite (version 4.2.1),^52^ using the relativistic DKH2 method^53,54^ with the all-electron old-DKH-TZVPP basis set implemented in ORCA. The latter was obtained by recontraction of the TZVPPAll basis set.^55^ We selected the TPSSh functional^56^ for NMR shielding calculations, as it was shown in previous studies to provide good results.^57^ Shielding tensors were obtained with the gauge-including atomic orbitals (GIAO) method.^58,59^ The resolution of identity and chain of spheres exchange (RIJCOSX) approximation was used in these calculations, with the size of the COSX grid set with the GridX6 and NoFinalGridX keywords. Auxiliary basis sets were generated with the Autoaux procedure.^60,61^ Bulk solvent effects (water) were considered with a continuum model of the solvent defined by the bulk dielectric constant and atomic surface tensions (SMD).^62^

## Supporting Information Available

NMRD and ^17^O data; relaxometric titration; ^1^H NMR spectrum of the ligand; and Cartesian coordinates obtained with DFT calculations (PDF)
