# Supplementary material for: Surprising Complexity of the [Gd(AAZTA)(H2O)2]− Chelate Revealed by NMR in the Frequency and Time Domains
Source: Inorg Chem. 2021 Dec 10;61(1):496–506. doi: 10.1021/acs.inorgchem.1c03194 (PMC8753608; doi:10.1021/acs.inorgchem.1c03194)
Supplement: Supplementary file 1 — ic1c03194_si_001.pdf [file ic1c03194_si_001.pdf]

Supporting Information

Surprising Complexity of the $[Gd(AAZTA)(H_2O)_2]^-$ Chelate Revealed by NMR in the Frequency and Time Domains

Daniela Lalli,*[a] Fabio Carniato,[a] Lorenzo Tei,[a] Carlos Platas-Iglesias,*[b] Mauro Botta[a,c]

[a] Dipartimento di Scienze e Innovazione Tecnologica, Università del Piemonte Orientale “A. Avogadro”, Viale T. Michel 11, 15121 Alessandria, Italy

[b] Centro de Investigacións Científicas Avanzadas (CICA) and Departamento de Química, Facultade de Ciencias, Universidade da Coruña, 15071, A Coruña, Galicia, Spain

[c] Magnetic Resonance Platform (PRISMA-UPO), Università del Piemonte Orientale “A. Avogadro”, Viale T. Michel 11, 15121 Alessandria, Italy

Email: daniela.lalli@uniupo.it (D.L.), carlos.platas.iglesias@udc.es (C.P.I.)

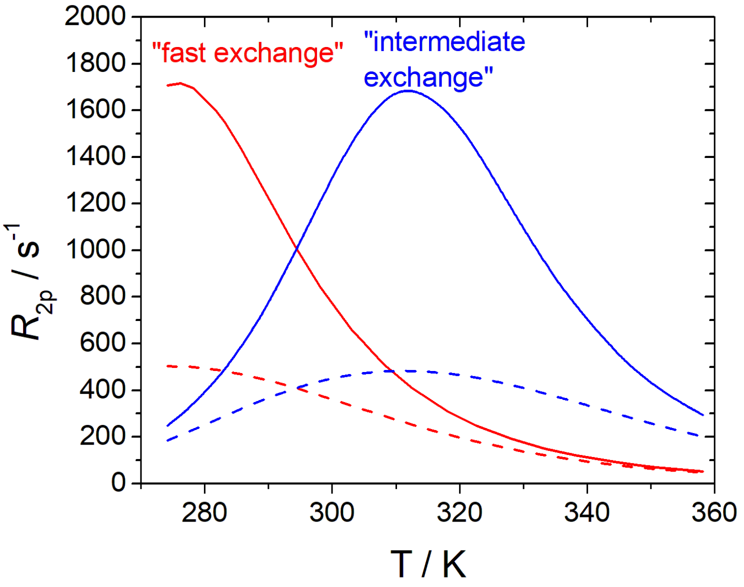

Figure S1. Temperature dependence of ^{17}O transverse relaxation rate calculated at 11.74 (solid lines) and 2.1 T (dashed lines) for a monohydrated Gd(III) complex 25 mM with τ_M = 30 ns, $\Delta H^\#$ = 40 kJ mol^{-1} (red profile) and τ_M = 300 ns, $\Delta H^\#$ = 48 kJ mol^{-1} (blue profile).

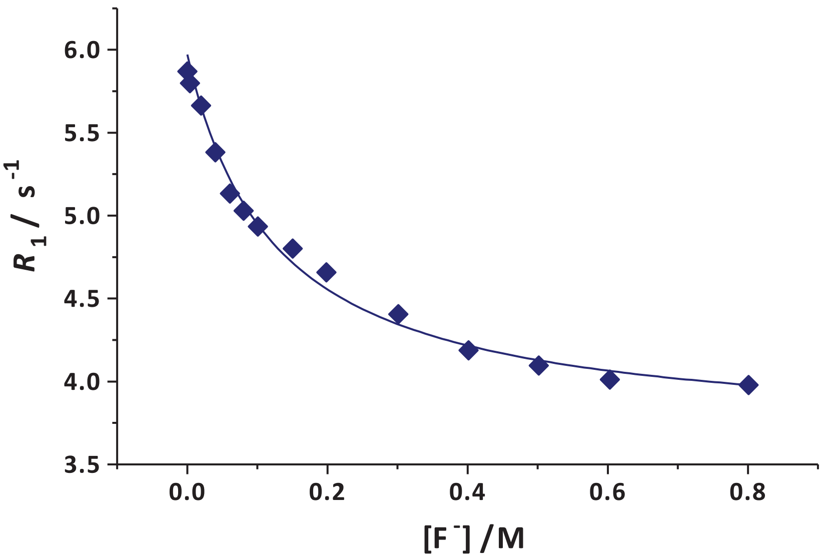

Figure S2. Relaxometric titration of 1.1 mM aqueous solution of $[Gd(AAZTA)(H_2O)_2]^-$ with increasing amounts of NaF (32 MHz, 310 K). The solid lines correspond to the fits of the data as described in the text.

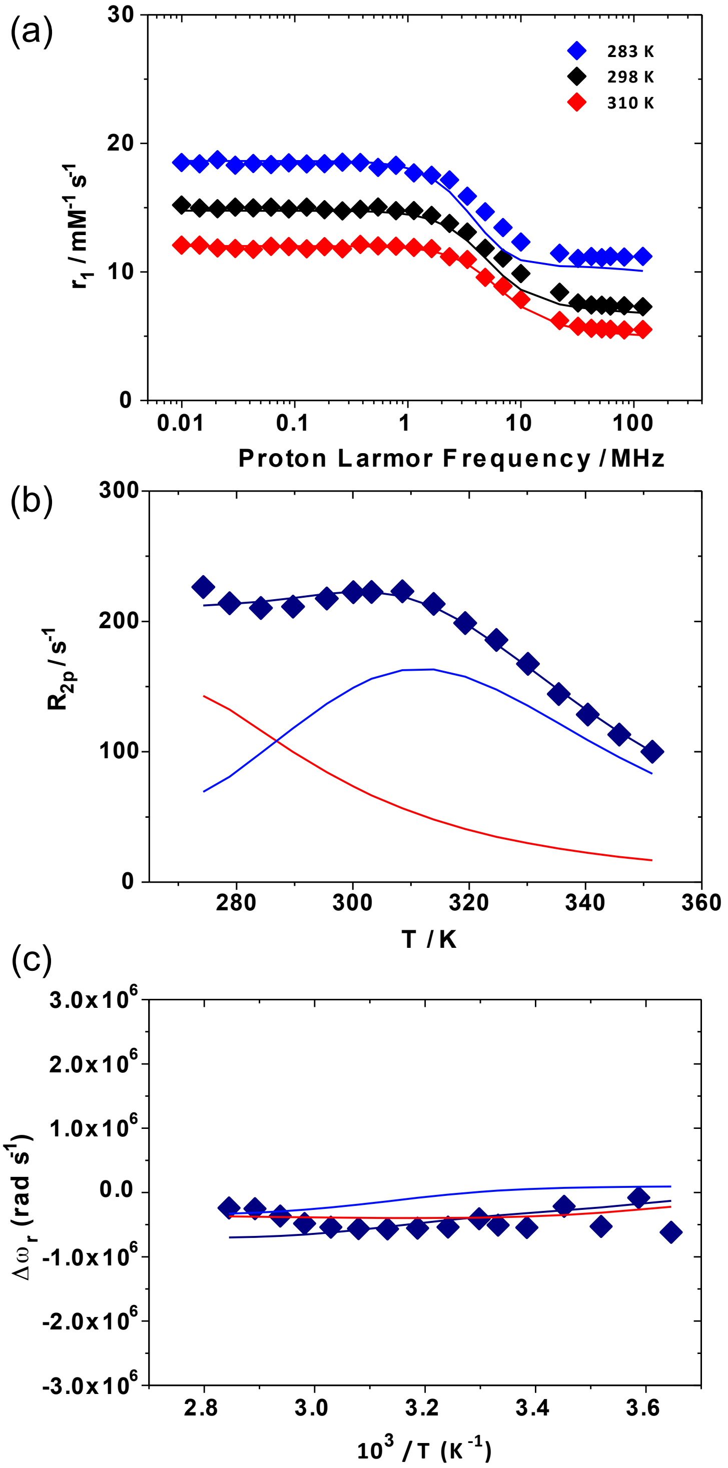

Figure S3. (a) ^{1}H NMRD profiles of a 1.0 mM solution of $[Gd(AAZTA)(H_2O)_2]^-$ in the presence of NaCl 600 mM, recorded at different temperatures 283 K (♦), 298 K (♦) and 310 K (♦). (b) ^{17}O transverse relaxation rates and (c) ^{17}O chemical shift variation (bottom) as a function of temperature of $[Gd(AAZTA)(H_2O)_2]^-$ 4.3 mM, in the presence of NaCl 600 mM, measured at 11.74 Tesla. The solid lines correspond to the fits of the data. The reported ^{1}H and ^{17}O NMR data are comparable with those of $[Gd(AAZTA)(H_2O)_2]^-$ in pure water, suggesting that the chloride anion does not coordinate to the metal centre, as showed in previous studies on $[Eu(DOTA)(H_2O)]^-$.[1]

Table S1. Parameters obtained from the fits of ^{1}H NMRD profiles and ^{17}O NMR data acquired at 11.74 Tesla for $[Gd(AAZTA)(H_2O)_2]^-$, $[Gd(AAZTA)(H_2O)F]^{2-}$ and $[Gd(AAZTA)(H_2O)_2]^-$ in the presence of 600 mM NaCl, in comparison with those obtained for the $[Gd(AAZTA)(H_2O)_2]^-$ with ^{17}O NMR data acquired at 2.1 T.[2]

	$[Gd(AAZTA)(H_2O)_2]^-$	$[Gd(AAZTA)(H_2O)F]^{2-}$	$[Gd(AAZTA)(H_2O)_2]^-$ 600 mM NaCl	$[Gd(AAZTA)(H_2O)_2]^-$(2)
$^{298}r_1$ / mM^{-1} s^{-1} (32 MHz)	6.6	4.2	7.0	6.6
$^{298}\Delta^2$ / 10^{19} s^{-2}	2.6	2.7	2.4	2.15
$^{298}\tau_V$ / ps	30	21	25	31
E_V / kJ mol^{-1}	1.0 [a]	1.0 [a]	1.0 [a]	-
$A_O^A/\hbar/10^6$ rad s^{-1}	-3.8	-	-3.8	-
$A_O^B/\hbar/10^6$ rad s^{-1}	-3.9	-3.8	-3.9	-3.8
$^{298}\tau_M^A$ / ns	29	-	29	-
ΔH_M^A / kJ mol^{-1}	20.0	-	23.0	-
$^{298}\tau_M^B$ / ns	169	3.4	188	90
ΔH_M^B / kJ mol^{-1}	29.5	23.4	28.0	-
$^{298}\tau_R$ / ps	74.0	74.0	74.0	74.0
E_R / kJ mol^{-1}	20.0	24.0	20.0	-
C_{os}	0.02	0.0	0.1	-
q	2 [a]	1 [a]	1 [a]	2 [a]
r / Å	3.05 [a]	3.05 [a]	3.05 [a]	3.05 [a]
a / Å	4.0 [a]	4.0 [a]	4.0 [a]	4.0 [a]
^{298}D / 10^5 cm^2 s^{-1}	2.24 [a]	2.24 [a]	2.24 [a]	2.24 [a]
E_D / kJ mol^{-1}	20.0 [a]	20.0 [a]	20.0 [a]	20.0 [a]

[a] Parameters fixed during the fitting procedure

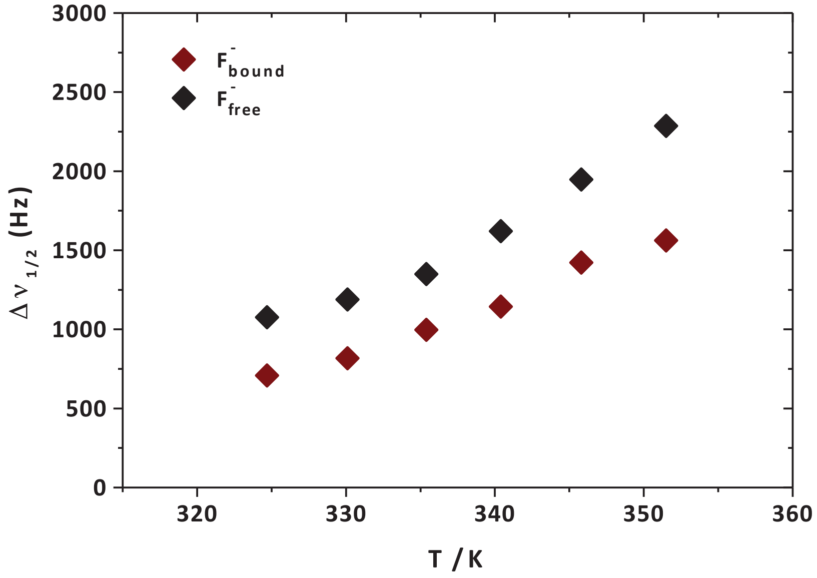

Figure S4. Line width variations of the ^{19}F NMR signals ($\Delta\nu_{1/2}(^{19}F)$) of the free (♦) and bound (♦) fluoride forms as a function of temperature, measured for the $[Y(AAZTA)(H_2O)F]^{2-}$ complex prepared as described in the Experimental Section. Line-shape analyses were carried out using the DNMR (DynamicNMR) LineShape Analysis tool (version 1.1.2) implemented in Bruker's Topspin 3.2. The DNMR Lineshape Fitting module simulates 1D temperature dependent NMR spectra of coupled half spin nuclei, iteratively refines the model parameters to get the best fit of the measured and simulated 1D NMR spectra, and once the best fit is reached, it provides the reaction rate parameters of the exchange processes, for each measured temperature. The theory of the calculation (density matrix theory for coupled spin systems), of the spectrum fitting and parameter refining (iterative methods) are described in the DNMR Lineshape Analysis software manual written by János Rohonczy, 2007. The method was adapted from the author's program TEDDY.[3]

Table S2. Kinetic parameters derived from Eyring plots for the exchange between the fluoride-bound and free forms.

**Δ*T* (K)**	***k* (325 K) (s^{-1})**	**$\Delta H^{\#}$ (kJ mol^{-1})**	**$\Delta S^{\#}$ (J K^{-1} mol^{-1})**	**$\Delta G^{\#}$ (325 K) (kJ mol^{-1})**
**325-350**	**325-350**	**32.3**	**-82.2**	**59.0**

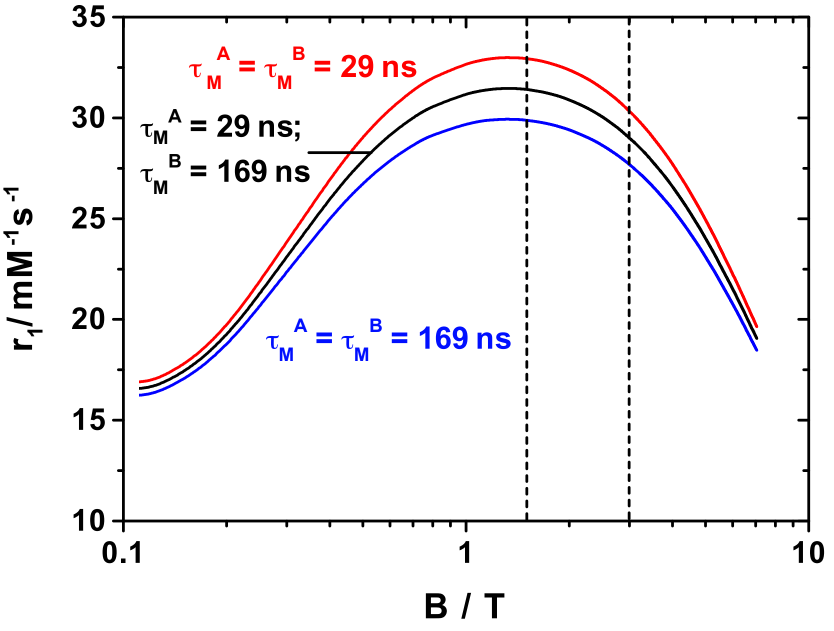

Figure S5. Relaxivities calculated for a bis-hydrated Gd(III) complex with a τ_R = 0.5 ns and different water residence times. All other parameters are those listed for $[Gd(AAZTA)(H_2O)_2]^-$ in Table 1. Vertical dashed lines indicate magnetic fields of 1.5 and 3 T.

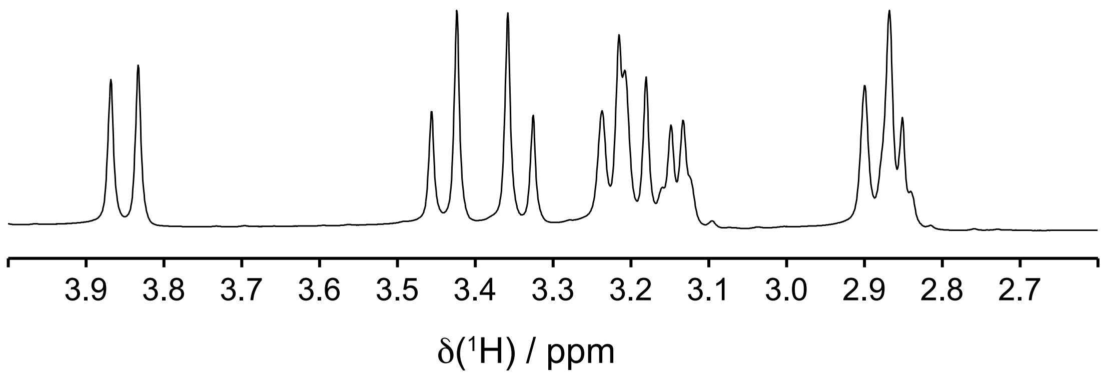

Figure S6. ^{1}H 1D NMR spectrum acquired on a 25 mM $[Y(AAZTA)(H_2O)_2]^-$ complex at 300 K, 11.4 Tesla. The complex was prepared as reported in the experimental section.

Table S3. Optimized Cartesian coordinates obtained for $[Gd(AAZTA)(H_2O)F]^{2-}$, where F^- replaces the more labile water molecule, (M062X/ECP53MWB/6-311G(d,p), scrf=(pcm,solvent=water)).

Center Number	Atomic Number	Coordinates (Å) X	Y	Z
1	64	0.221536	0.351938	-0.276800
2	8	-1.799373	1.121915	-1.416951
3	7	-1.335141	-1.710974	0.201823
4	6	-0.528715	-2.956268	0.245606
5	1	-1.052885	-3.773740	-0.255309
6	1	-0.357429	-3.278030	1.272333
7	8	-3.220283	2.832832	-1.623439
8	7	0.092715	-0.375306	2.338762
9	6	0.868313	-2.802012	-0.379229
10	7	-1.861918	1.144866	1.268958
11	6	-2.048737	-1.389095	1.482254
12	6	-2.759201	-0.027469	1.354461
13	1	-3.404471	-0.026338	0.478079
14	1	-3.424166	0.081038	2.222167
15	6	-1.422650	1.572304	2.611673
16	1	-2.208906	1.358844	3.345441
17	1	-1.276609	2.651644	2.607454
18	8	1.316622	-3.736172	-1.053319
19	8	1.475470	-1.723768	-0.105971
20	6	-0.100900	0.916051	3.017388
21	1	-0.071970	0.782439	4.107977
22	1	0.731344	1.555981	2.727950
23	8	-0.292404	-1.067229	-2.216233
24	6	-1.018898	-1.300467	2.631961
25	1	-1.555742	-0.984118	3.534583
26	1	-0.620967	-2.287260	2.864675
27	8	-1.869054	-2.219872	-3.302519
28	6	-2.205001	-1.809096	-0.986481
29	1	-2.902446	-0.977068	-1.030429
30	1	-2.773695	-2.743969	-1.000177
31	6	-1.396999	-1.710005	-2.292069
32	6	-2.529713	2.240738	0.560017
33	1	-1.954153	3.154566	0.716816
34	1	-3.552378	2.412479	0.918877
35	8	2.164336	0.737400	1.102856
36	6	-2.541305	2.041658	-0.958157
37	8	3.700404	-0.518097	2.107051
38	6	-3.097115	-2.448888	1.835278
39	1	-3.920671	-2.436546	1.118056
40	1	-2.654822	-3.447725	1.833497
41	6	1.418647	-0.904089	2.677945
42	1	1.482089	-1.949655	2.379963

43	1	1.624873	-0.840521	3.755015
44	6	2.526977	-0.170629	1.907481
45	8	2.189824	0.541808	-1.797665
46	1	-3.511603	-2.263584	2.828253
47	1	2.286564	-0.322850	-2.250069
48	1	2.991525	0.629812	-1.244585
49	8	-0.612813	4.109708	-2.120818
50	1	-0.258853	3.611146	-1.361430
51	1	-1.525123	3.801297	-2.187008
52	8	3.055988	3.223714	-0.198940
53	1	2.118033	2.987017	-0.101227
54	1	3.514609	2.391525	-0.025080
55	8	4.552472	0.681554	-0.264634
56	8	2.114205	-1.909012	-3.108643
57	1	5.420630	0.387852	-0.551221
58	1	4.367203	0.249197	0.595862
59	1	2.186742	-2.586763	-2.416241
60	1	1.164867	-1.684319	-3.044782
61	9	0.388792	2.610991	-0.116222

E(RM062X) = -1829.4238761 Hartree
Zero-point correction = 0.482587
Thermal correction to Energy = 0.520166
Thermal correction to Enthalpy = 0.521110
Thermal correction to Gibbs Free Energy = 0.415882
Sum of electronic and zero-point Energies = -1828.941289
Sum of electronic and thermal Energies = -1828.903710
Sum of electronic and thermal Enthalpies = -1828.902766
Sum of electronic and thermal Free Energies = -1829.007994

Table S4. Optimized Cartesian coordinates obtained for $[Gd(AAZTA)(H_2O)F]^{2-}$, where F^- replaces the more less water molecule, (M062X/ECP53MWB/6-311G(d,p), scrf=(pcm,solvent=water)).

Center Number	Atomic Number	Coordinates (Å) X	Y	Z
1	64	0.258432	0.258340	-0.344746
2	8	-1.646462	1.396926	-1.328503
3	7	-1.507060	-1.629605	0.167718
4	6	-0.832254	-2.950129	0.117850
5	1	-1.438092	-3.672800	-0.433958
6	1	-0.693626	-3.360212	1.118312
7	8	-2.780909	3.313426	-1.416825
8	7	0.127405	-0.481905	2.293562
9	6	0.571459	-2.899756	-0.507281
10	7	-1.754162	1.227285	1.359164

11	6	-2.123671	-1.295320	1.493497
12	6	-2.728403	0.120039	1.443955
13	1	-3.410285	0.200889	0.600075
14	1	-3.346825	0.245282	2.343786
15	6	-1.231611	1.560685	2.697367
16	1	-1.999916	1.370406	3.456644
17	1	-1.003337	2.624846	2.735214
18	8	0.937937	-3.837635	-1.218009
19	8	1.277926	-1.892608	-0.184553
20	6	0.052707	0.796233	3.017020
21	1	0.121549	0.626582	4.101147
22	1	0.913767	1.388644	2.710033
23	8	-0.509860	-1.010847	-2.262317
24	6	-1.036079	-1.337326	2.591749
25	1	-1.505254	-1.036799	3.537161
26	1	-0.699189	-2.361514	2.747754
27	8	-2.257562	-1.923216	-3.320574
28	6	-2.435189	-1.580281	-0.978464
29	1	-3.026135	-0.667784	-0.963236
30	1	-3.115560	-2.437402	-0.993802
31	8	0.592833	2.716952	0.009631
32	6	-1.673155	-1.519329	-2.315445
33	6	-2.372913	2.392962	0.722723
34	1	-1.823663	3.288862	1.011485
35	1	-3.418228	2.535253	1.025480
36	8	2.232811	0.522357	1.038458
37	6	-2.283631	2.356467	-0.804191
38	8	3.713991	-0.823586	2.010867
39	6	-3.241378	-2.275407	1.868009
40	1	-4.093889	-2.168766	1.193717
41	1	-2.889348	-3.307859	1.814670
42	6	1.417708	-1.114618	2.582381
43	1	1.402907	-2.147247	2.237877
44	1	1.648532	-1.111053	3.656546
45	6	2.563267	-0.427015	1.827216
46	1	-3.590810	-2.089083	2.885501
47	1	0.364650	3.246358	-0.782155
48	1	1.518282	2.937840	0.242453
49	8	-0.243919	4.004929	-2.310827
50	1	0.054937	3.456217	-3.041763
51	1	-1.198194	3.816898	-2.226964
52	8	3.256872	2.964209	0.600907
53	1	3.453008	3.279701	1.487505
54	1	3.197868	1.991415	0.689705
55	8	4.451587	0.169567	-0.741344
56	8	2.550887	-1.891075	-2.897228
57	1	3.556538	0.360563	-1.070866
58	1	4.292609	-0.103570	0.172662
59	1	2.111677	-2.518893	-2.307403

60	1	2.295079	-1.033005	-2.520472
61	9	1.914198	0.629293	-1.831358

E(RM062X) = -1829.4151223 Hartree
Zero-point correction = 0.481837
Thermal correction to Energy = 0.519969
Thermal correction to Enthalpy = 0.520913
Thermal correction to Gibbs Free Energy = 0.414447
Sum of electronic and zero-point Energies = -1828.933285
Sum of electronic and thermal Energies = -1828.895153
Sum of electronic and thermal Enthalpies = -1828.894209
Sum of electronic and thermal Free Energies = -1829.000676

Table S5. Optimized Cartesian coordinates obtained for $[Y(AAZTA)(H_2O)F]^{2-}$ (M062X/ ECP28MWB/6-311G(d,p), scrf=(pcm,solvent=water)).

Center Number	Atomic Number	Coordinates (Å) X	Y	Z
1	39	-0.269186	0.352629	0.222884
2	8	1.492772	1.429836	1.374676
3	7	1.522985	-1.490475	-0.004731
4	6	0.871268	-2.823319	0.013183
5	1	1.450348	-3.531850	0.610105
6	1	0.805848	-3.244291	-0.989670
7	8	2.524641	3.397176	1.573566
8	7	0.079646	-0.513341	-2.304803
9	6	-0.570902	-2.785016	0.539947
10	7	1.760223	1.307597	-1.274612
11	6	2.270790	-1.185567	-1.269112
12	6	2.800693	0.260066	-1.216930
13	1	3.383140	0.408838	-0.309752
14	1	3.501411	0.388561	-2.053476
15	6	1.361064	1.576123	-2.668988
16	1	2.210190	1.403246	-3.341019
17	1	1.089840	2.626585	-2.765263
18	8	-0.973996	-3.730130	1.228380
19	8	-1.259522	-1.785501	0.177927
20	6	0.154020	0.732946	-3.081831
21	1	0.203482	0.510780	-4.157330
22	1	-0.763524	1.286354	-2.888240
23	8	0.263426	-0.784258	2.273444
24	6	1.306835	-1.314444	-2.471213
25	1	1.852955	-1.003077	-3.369969
26	1	1.044746	-2.357935	-2.641843
27	8	1.883777	-1.634791	3.554791
28	6	2.325209	-1.395539	1.230756

29	1	2.932421	-0.494943	1.237878
30	1	2.984035	-2.260452	1.356020
31	6	1.426617	-1.276451	2.473093
32	6	2.228270	2.527316	-0.610554
33	1	1.583926	3.354473	-0.908603
34	1	3.260694	2.784923	-0.881015
35	8	-2.130232	0.434761	-1.213815
36	6	2.093101	2.446046	0.911202
37	8	-3.494811	-1.025097	-2.188004
38	6	3.457491	-2.131123	-1.477554
39	1	4.226950	-1.964055	-0.720563
40	1	3.138061	-3.174133	-1.418455
41	6	-1.160411	-1.215472	-2.648256
42	1	-1.120335	-2.238916	-2.277542
43	1	-1.329263	-1.251181	-3.733202
44	6	-2.368391	-0.552525	-1.968573
45	8	-2.231224	0.493303	1.641107
46	1	3.908540	-1.969981	-2.458836
47	1	-2.283388	-0.344910	2.144889
48	1	-3.015037	0.478640	1.057627
49	8	-0.320380	3.792719	2.271091
50	1	-0.500117	3.367084	1.413520
51	1	0.636992	3.690719	2.354093
52	8	-3.393968	2.895568	-0.085219
53	1	-2.440626	2.713870	-0.148208
54	1	-3.798405	2.026617	-0.210553
55	8	-4.569353	0.239753	0.056781
56	8	-2.048440	-1.864297	3.138662
57	1	-5.411202	-0.125555	0.339597
58	1	-4.303069	-0.233155	-0.760375
59	1	-2.017656	-2.585208	2.488441
60	1	-1.130136	-1.531564	3.089478
61	9	-0.695331	2.482324	-0.068810

E(RM062X) = -1831.8740618 Hartree
Zero-point correction = 0.483582
Thermal correction to Energy = 0.520538
Thermal correction to Enthalpy = 0.521482
Thermal correction to Gibbs Free Energy = 0.419438
Sum of electronic and zero-point Energies = -1831.390480
Sum of electronic and thermal Energies = -1831.353524
Sum of electronic and thermal Enthalpies = -1831.352580
Sum of electronic and thermal Free Energies = -1831.454624

References:

1. Aime, S.; Botta, M.; Fasano, M.; Marques, M. P. M.; Geraldes, C. F.; Pubanz, D.; Merbach, A. E. Conformational and coordination equilibria on DOTA complexes of lanthanide metal ions in aqueous solution studied by ^{1}H-NMR spectroscopy. *Inorg. Chem.* **1997,** 36 (10), 2059-2068.
2. Aime, S.; Calabi, L.; Cavallotti, C.; Gianolio, E.; Giovenzana, G. B.; Losi, P.; Maiocchi, A.; Palmisano, G.; Sisti, M. [Gd-AAZTA]-: a new structural entry for an improved generation of MRI contrast agents. *Inorg. Chem.* **2004,** 43 (24), 7588-7590.
3. Rohonczy, J. Total lineshape analysis of DNMR spectra by IBM personal computer. *Kem. Kozl* **1992,** 74, 161-200.
